# Bio-Inspired Amphiphilic Block-Copolymers Based on Synthetic Glycopolymer and Poly(Amino Acid) as Potential Drug Delivery Systems

**DOI:** 10.3390/polym12010183

**Published:** 2020-01-10

**Authors:** Mariia Levit, Natalia Zashikhina, Alena Vdovchenko, Anatoliy Dobrodumov, Natalya Zakharova, Anna Kashina, Eckart Rühl, Antonina Lavrentieva, Thomas Scheper, Tatiana Tennikova, Evgenia Korzhikova-Vlakh

**Affiliations:** 1 Institute of Macromolecular Compounds, Russian Academy of Sciences, Bolshoy pr. 31, 199004 St. Petersburg, Russia; musia_1@yahoo.com (M.L.); nzashihina@bk.ru (N.Z.); anatoliy.dob@gmail.com (A.D.); or na_zar@inbox.ru (N.Z.); kashina.anna@mail.ru (A.K.); 2 Institute of Chemistry, Saint-Petersburg State University, Universitetsky pr. 26, 198504 St. Petersburg, Russia; vdovchjenko@yandex.ru (A.V.); tennikova@mail.ru (T.T.); 3 Physical Chemistry, Institute of Chemistry and Biochemistry, Freie Universität Berlin, 14195 Berlin, Germany; ruehl@zedat.fu-berlin.de; 4 Institute of Technical Chemistry, Gottfried-Wilhelm-Leibniz University of Hannover, 30167 Hannover, Germany; lavrentieva@iftc.uni-hannover.de (A.L.); scheper@iftc.uni-hannover.de (T.S.)

**Keywords:** synthetic glycopolymers, poly(amino acids), amphiphilic block copolymers, modification of terminal groups, nanoparticles, redox-responsive systems, paclitaxel, drug delivery systems

## Abstract

In this work, a method to prepare hybrid amphiphilic block copolymers consisting of biocompatible synthetic glycopolymer with non-degradable backbone and biodegradable poly(amino acid) (PAA) was developed. The glycopolymer, poly(2-deoxy-2-methacrylamido-D-glucose) (PMAG), was synthesized via reversible addition-fragmentation chain transfer (RAFT) polymerization. Two methods for modifying the terminal dithiobenzoate-group of PMAG was investigated to obtain the macroinitiator bearing a primary aliphatic amino group, which is required for ring-opening polymerization of *N*-carboxyanhydrides of hydrophobic α-amino acids. The synthesized amphiphilic block copolymers were carefully analyzed using a set of different physico-chemical methods to establish their composition and molecular weight. The developed amphiphilic copolymers tended to self-assemble in nanoparticles of different morphology that depended on the nature of the hydrophobic amino acid present in the copolymer. The hydrodynamic diameter, morphology, and cytotoxicity of polymer particles based on PMAG-*b*-PAA were evaluated using dynamic light scattering (DLS) and transmission electron microscopy (TEM), as well as CellTiter-Blue (CTB) assay, respectively. The redox-responsive properties of nanoparticles were evaluated in the presence of glutathione taken at different concentrations. Moreover, the encapsulation of paclitaxel into PMAG-*b*-PAA particles and their cytotoxicity on human lung carcinoma cells (A549) and human breast adenocarcinoma cells (MCF-7) were studied.

## 1. Introduction

Currently, the development of polymer nanoparticles as drug delivery and detection systems has been extensively investigated [1,2,3]. The main advantages of polymer systems compared to inorganic materials are the variety of their structure and properties, the higher drug loading capacities, and the diversity of reactive functional groups for the attachment of biomolecule-vectors. Additionally, contrary to the many inorganic materials, having the tendency to accumulate in the body, most of the polymer systems are characterized by predictable degradation mechanisms.

Among the wide range of biocompatible and biodegradable polymers utilized for preparation of nanoparticles, amphiphilic copolymers allow for the simple formation of nanostructures of different morphology (polymersomes, micelles, nanospheres) due to their ability to self-assemble in aqueous media [4,5,6]. Self-assembled nanoparticles are suitable for encapsulation of hydrophobic, hydrophilic and amphiphilic drugs [7,8,9]. Nowadays, a wide variety of amphiphilic copolymers of different structure and composition have been described and discussed [4,10,11]. Poly(ethylene glycol) (PEG), poly(ethylene oxide) (PEO), polysaccharides (heparin, chitosan, alginate, etc.), hydrophilic poly(amino acids) are usually used as hydrophilic blocks, whereas polyesters (poly(lactic acid) (PLA), polycaprolactone, etc.), polycarbonates, hydrophobic poly(amino acids), polystyrene, and poly(butyl methacrylate) are considered as hydrophobic ones. Despite the fact that numerous amphiphilic copolymers are known, only a limited number of them may be considered as suitable candidates for drug delivery. To be safe, the nanoparticles proposed for in vivo application have to be biocompatible, preferably biodegradable, and the mechanism of their elimination from the body should be predictable.

The combination of non-degradable or partly degradable polymers with biodegradable ones allows for the improvement of stability of polymer nanoparticles in vivo. This is especially important for the development of drug delivery systems with prolonged action. Some of “hybrid” copolymers, consisting of non-degradable and degradable blocks, were described for the preparation of self-assembled nanoparticles for drug delivery. In particular, poly(ethylene glycol)-*b-*poly(ε-caprolactone) [12,13], poly(ethylene glycol)-*b*-poly(D,L-lactide) [14], polybutadiene-*b*-poly(L-glutamate) [15], polybutadiene-*b*-poly(L-lysine) [16], and poly(L-Z-lysine)-*b*-poly(ethylene glycol)-*b*-poly(L-Z-lysine) [17] were reported for the preparation of polymersomes and polymer micelles. 

One of the promising classes of polymers in a view of its biomedical application are *glycopolymers* [18]. These are hydrophilic bio-inspired polymers with biodegradable or non-biodegradable carbon–carbon backbone bearing side saccharide units. In general, glycopolymers can be prepared by post-polymerization glycosylation of different synthetic polymers [19,20,21] or by ring-opening polymerization (ROP) of glycosylated α-amino acid *N*-carboxyanhydrides (NCAs) [22,23]. Well-defined glycopolymers can be synthesized by one of the approaches of controlled polymerization, such as nitroxide-mediated radical polymerization (NMP), atom transfer radical polymerization (ATRP), reversible addition-fragmentation chain-transfer (RAFT) polymerization, etc. [24,25,26]. The polymerization of glycomonomers provides the formation of homopolymers with predetermined structure, whereas post-polymerization glycosylation results in the formation of random copolymers consisting of glycosylated and non-glycosylated monomer units. Both approaches have their strong and week sides and can be a matter of choice depending on the final goal.

The presence of natural saccharide units makes synthetic glycopolymers to be biocompatible and capable to molecular recognition towards lectins [27]. In addition, glycopolymers with neutral saccharide units enhance the stability of nanoparticles with respect to the uptake by macrophages [28]. The partial oxidation of vicinal hydroxyl groups of saccharide units allows for the introduction of highly reactive aldehydes that are very useful for conjugation with amino-bearing biomolecules or labels [29]. In spite of successful synthesis of homoglycopolymers [26,30,31,32] and their block copolymers with other non-degradable polymers [33,34,35], only a few works on the synthesis of block copolymers consisted of hydrophilic glycopolymers with non-degradable backbone and biodegradable hydrophobic blocks were reported [36,37,38]. The main challenge in the preparation of amphiphilic glycopolymer-*b*-polyester or glycopolymer-*b*-poly(amino acid) is the combination of different polymerization techniques. Ting et al. reported the synthesis of poly(6-*O*-acryloyl-α-d-galactopyranose)-*b*-poly(lactic acid) via combination of RAFT and ROP techniques [36]. In the described approach, the block-copolymer was synthesized using modified PLA as macroRAFT agent. Dong et al. described the synthesis of poly(L-glutamate)-poly(2-acryloyl oxyethyl lactoside)-poly(L-glutamate) [39] and poly(L-alanine)-*b*-poly(2-acryloyl oxyethyl lactoside)-*b*-poly(L-alanine) [40] based on the preparation of poly(2-acryloyl oxyethyl lactoside) via ATRP and modification of glycopolymer terminal groups bearing bromine with tert-butoxycarbonyl-1,4-diaminobutane with further removal of the tert-butoxycarbonyl (BOC) protective group followed by ROP of *N*-carboxyanhydrides of α-amino acids. In other works, the amphiphilic copolymers (poly(allyl glycidyl ether-glucose)-*b*-poly(lactic acid) and poly(caprolactone-glucose)-*b*-poly(ε-caprolactone)) were prepared using post-modification of hydrophobic block-copolymer via click-reaction with sugar derivatives [37,38].

The first attempt to synthetize the glycopolymer-*b*-poly(amino acid) using the combination of RAFT polymerization and ROP was recently fulfilled by our group. Well-defined poly(2-deoxy-2-metacryloylamido-D-glucose) (PMAG) was synthesized via RAFT polymerization and its terminal groups were modified to prepare macroinitiator for ROP of *N*-carboxyanhydride of phenylalanine [41]. In this work, we continued to develop the procedures for the synthesis of “hybrid” copolymers based on PMAG and poly(amino acids) and we focused on the optimization of methods for the preparation of macroinitiators and copolymers with certain chemical composition, and ability to selective degradation. The synthesis of two novel copolymers, e.g., PMAG-*b*-poly(L-isoleucine) (PMAG-b-PIle) and PMAG-*b*-poly(γ-benzyl L-glutamate) (PMAG-b-PGlu(OBzl)), as well as the preparation of nanoparticles based on these copolymers were carried out. The designed copolymers have a great potential as drug delivery systems. First of all, the application of PMAG as a hydrophilic fragment can provide biocompatibility of nanoparticles due to its bio-inspired nature, as well as higher stability to degradation compared to fully biodegradable systems, such as poly(amino acids). The latter property is very important to prevent the premature degradation of delivery systems in the blood stream. Second, linking of hydrophilic and hydrophobic blocks via a disulfide bond enhances intracellular degradation by glutathione with intracellular concentration to a much higher degree than extracellular degradation. Finally, the water-soluble PMAG can be eliminated from the body via kidney filtration, whereas hydrophobic poly(amino acid) blocks will degrade up to free amino acids.

To evaluate the potential of developed nanoparticles based on amphiphilic PMAG-b-PAA as drug delivery systems, the characteristics and morphology of these nanoparticles depending on the polymer composition, as well as their biodegradability, stability, and cytotoxicity were established. Finally, the encapsulated forms of paclitaxel based on the developed polymer particles were obtained and the in vitro anti-tumoral activity of paclitaxel-loaded particles on A549 (human lung carcinoma cells) and MCF-7 (human breast adenocarcinoma) cancer cells was evaluated.

## 2. Materials and Methods 

### 2.1. Materials

γ-benzyl-L-glutamate (Glu(OBzl)) (>99%), L-isoleucine (Ile) (>98%), triphosgene (98%), α-pinene (98%), 2-aminoethanethiol hydrochloride (AETL) (cysteamine hydrochloride) (>98%), 2-aminoethyl methacrylate hydrochloride (AEMA) (90%), *n*-hexylamine (HexNH_2_, 98%), sodium borohydride (>96%), 4-cyanopentanoic acid-4-dithiobenzoate (CTA, >97%), 2,4,6-trinitrobenzenesulfonic acid (TNBS) (>98%) were purchased from Sigma-Aldrich (Darmstadt, Germany) and used as received. 2,2′-Azobisisobutyronitrile (AIBN, Acros Organics, Geel, Belgium, 98%) was purified by recrystallization from methanol and dried under vacuum. *N*,*N*-dimethylformamide (DMF), dimethyl sulfoxide (DMSO), 1,4-dioxane, ethyl acetate, methanol, chloroform, *n*-hexane, triethylamine (TEA), and other solvents used in this work were purchased from Vecton Ltd. (St. Petersburg, Russia) and distilled before use. 

Membranes for dialysis with molecular weight cut-off (MWCO) 1000, 2000, 3500, 6000–8000, and 12,000–14,000 were purchased from Orange Scientific (OrDialDClean regenerated cellulose dialysis tubing, Anaheim, CA, USA). Vivaspin concentrators used for ultrafiltration were products of Sartorius (Göttingen, Germany).

Human embryonic kidney cells (HEK 293), human lung carcinoma cells (A549), and human bronchial epithelial cells (BEAS-2B) were purchased from CLS Cell Lines Service GmbH (Eppelheim, Germany). The first three cell lines were cultivated in Dulbecco’s Modified Eagle’s Medium (Merck, Darmstadt, Germany) and the last one in LHC-9 (Thermo Fisher Scientific, Dreieich, Germany) medium, supplemented with 10% (*v*/*v*) fetal calf serum (FCS) (Biochrom, Berlin, Germany), and 1% (*v*/*v*) penicillin/streptomycin (P/S) (Biochrom, Berlin, Germany). 

MCF-7 (human breast adenocarcinoma) cells were purchased from German Collection of Microorganisms and Cell Culture (Braunschweig, Germany) and grown in Modified Eagle’s Medium (Merk, Germany) containing 10% (*v*/*v*) FCS (Biochrom, Germany), 1% L-glutamine, 1% sodium pyruvate, 50 U/mL penicillin, and 50 μg/mL streptomycin (Biochrom, Berlin, Germany), 0.1% nonessential amino acids (NEA) (Biochrom, Berlin, Germany), and 1 μM insulin (Merck, Darmstadt, Germany). CellTiter-Blue cell viability assay reagent was purchased from Promega (Madison, WI, USA).

### 2.2. Synthesis of Monomers

2-Deoxy-2-methacrylamido-D-glucose (MAG) was synthesized according to previously published protocol [41]. Ile and Glu(OBzl) NCAs were synthesized by phosgenation of amino acid with the use of triphosgene as a precursor using the protocol described elsewhere [42]. Dioxane was used as a solvent for Ile, whereas THF was the solvent for Glu(OBzl) synthesis. Acquired NCAs were purified by recrystallization from ethyl acetate/*n*-hexane. Yields: Glu(OBzl) NCA—79%, Ile NCA—70%. ^1^H nuclear magnetic resonance (NMR) spectroscopy of Glu(OBzl) NCA (CDCl_3_, 400 MHz), δ (ppm): 2.03–2.23 (m, 1H, CH_2_), 2.23–2.37 (m, 1H, CH_2_), 2.62 (t, *J* = 6.8 Hz, 2H, CH_2_), 4.40 (t, *J* = 6.2 Hz, 1H, CH), 5.16 (s, 2H, C_6_H_5_OCH_2_), 6.66 (s, 1H, NH), 7.28–7.45 (m, 5 H, C_6_H_5_). ^1^H NMR of Ile NCA (CDCl_3_, 400 MHz), δ (ppm): 1.0 (3H, d, -C-CH_3_), 1.1–1.9 (6 H, m, -CH-CH_2_-CH_3_), 4.3 (1H, s, CH(NHR)-COOR), 6.2 (1H, s, NH). 

### 2.3. Synthesis of PMAG-CTA 

The synthesis of PMAG-CTA was carried out via RAFT polymerization at 70 °C for 16 h in a Schlenk flask using AIBN, CTA, and DMF as initiator, RAFT chain transfer agent and solvent, respectively. The initial ratios of [MAG]_o_:[CTA]_o_:[AIBN]_o_ equal to 20:1:0.25 and 75:1:0.25 were applied. Before polymerization the mixture was degassed via four freeze-evacuated-thaw cycles, refilled with argon and then immersed in a preheated oil bath. The product was isolated by precipitation into excess of diethyl ether and dried under vacuum. The monomer conversion was determined by ^1^H NMR spectroscopy in D_2_O by comparing the relative integral areas of five protons of CH_3_ and CH_2_ groups of the polymer (0.8–2.0 ppm) or 6 protons of glucose rings (3.4–4.0 ppm) to the vinyl protons from unsaturated bonds of the monomer (5.48 or 5.71 ppm). 

For calculation of monomer conversion (*x*) the following equation was used:

*x =* (1 − (*I*(*MAG*)_5.48 ppm_*/I*(*MAG + PMAG*)_3.4–4.0 ppm_)) × 100% (1)

where *I*(*MAG*)_5.48 ppm_ and *I*(*MAG + PMAG*)_3.4–4.0 ppm_ correspond to the integral intensity of MAG protons at 5.48 ppm and MAG + PMAG at 3.4–4.0 ppm, respectively.

To remove unreacted monomers and other impurities, the prepared polymer was dialyzed against deionized water (MWCO 2000) and lyophilized. The yield of PMAG-CTA, a light pink powder, was 63–75%.

According to NMR spectroscopy (D_2_O, 400 MHz), δ (^1^H,^13^C) (ppm) the signals of protons of MAG units, namely: -CH_3_ (0.93–1.39; 16.2–20.7), -CH_2_-C- (1.39–2.11; 54.76), glucose ring C_2_H_2_-C_6_H_6_ (3.4–4.0; 55–77), C_1_H_1_ α (5.03–5.30; 91.2) and β (4.78; 95.9), as well as signals of the R group -CH_2_-CH_2_-COOH (2.55; 30.5), -CH_2_-CH_2_-COOH (1.88, 1.98; 37.5), and aromatic protons of Z groups at o-, (7.52; 129.7) p-(7.66, 134.2) and m-positions (7.88, 127.6) of CTA, were detected. 

Theoretical molar weights *M_n_^th^* were calculated by the following equation:

*M_n_^th^* = *M_MAG_* × ([*MAG*]_*o*_/[*CTA*]_*o*_)*x* + *M_CTA_* (2)

where *M_MAG_*, *M_CTA_*, [*MAG*]*_o_*, and [*CTA*]*_o_* are the molar weights of MAG and CTA, and initial concentrations of MAG and CTA, respectively; *x* is the fractional conversion of MAG.

### 2.4. Modification of PMAG-CTA with 2-Aminoethanethiol Hydrochloride (AETL)

To introduce the primary amino group, necessary for polymerization of α-amino acid NCAs, the disulfide-linked amino functionalized PMAG was prepared from PMAG-CTA by reduction with NaBH_4_ or aminolysis (a mixture of triethylamine and hexylamine), followed by in situ reaction of the obtained thiol group with 2-aminoethanethiol hydrochloride with the formation of disulfide bond. 

Reduction with NaBH_4_. The molar ratio [PMAG-CTA]:[NaBH_4_]:[AETL·HCl] = 1:100:180 was used. Before the addition of NaBH_4,_ the solution containing PMAG-CTA in DMF (or DMF/H_2_O mixture) (5 wt%) was purged for 20 min with argon to remove the oxygen. NaBH_4_ in DMF (5.5 wt%) was added under stirring in inert atmosphere to reach 100-fold excess relative to the amount of dithiobenzoate groups (*mol*/*mol*). After few minutes, the pink color of the solution disappeared. The solution was stirred for 1 h and then AETL hydrochloride was added to reach 180-fold excess relative to amount of dithiobenzoate groups (*mol*/*mol*). For oxidation of the -SH groups and formation of disulfide bonds 0.2 M solution of I_2_ in DMF was added until the solution acquired color. The unreacted iodine was subsequently reduced by ascorbic acid. After 1 h the resulting mixture was dialyzed against deionized water, then the solution was concentrated by the use of a rotary evaporator. The product was isolated by precipitation into acetone and dried under vacuum. The yield of PMAG-SS(CH_2_)_2_NH_2_ was 79 ± 5%. In ^1^H NMR recorded in D_2_O at 60 °C the signals corresponding to CH_2_ group of AETL at 3.05 ppm (2H, -CH_2_-C*H*_2_-NH_2_) and 3.4 ppm (2H, -C*H*_2_-CH_2_-NH_2_) were detected.

Aminolysis with TEA and HexNH_2_. The molar ratio [PMAG-CTA]:[TEA]:[HexNH_2_]: [AETL·HCl] equal to 1:25:25:180 was used for PMAG-CTA modification. Briefly, PMAG-CTA was dissolved in DMF to reach a 5 wt% solution, which was purged for 30 min with argon to remove the oxygen. Then, the mixture of TEA and HexNH_2_ in DMF (0.1 M) was added under stirring in an inert atmosphere. The amounts of TEA and HexNH_2_ were taken to reach a 25 excess over the dithiobenzoate groups (*mol*/*mol*). After a few minutes, the pink color of the solution disappeared. The solution was stirred for 1 h and then a 180-fold excess of AETL hydrochloride was added to the reaction mixture. For oxidation of -SH groups along with the formation of disulfide bonds 1 M solution of I_2_ in DMF was added until the solution acquired color. The unreacted iodine was reduced by ascorbic acid. To remove the hydrochloride group of PMAG-NH_2_ the equimolar amount of TEA (0.15 mL) was added. After 1 h, the resulting mixture was dialyzed against deionized water and the solution was concentrated with the use of a rotary evaporator. The product was isolated by precipitation into acetone and dried under vacuum. The yield of PMAG-SS(CH_2_)_2_NH_2_ was equal to 70 ± 5%. The polymer characterization was done as described above for NaBH_4_ reduction. 

### 2.5. Modification of PMAG-CTA with 2-Aminoethyl Methacrylate Hydrochloride (AEMA)

PMAG bearing a terminal amino group was also prepared from PMAG-CTA by aminolysis following an in situ AEMA thiol-ene addition. The molar ratio [PMAG-CTA]:[TEA]:[HexNH_2_]:[AEMA·HCl] of 1:10:10:20 was used. PMAG-CTA was dissolved in DMF to reach an 8 wt% solution and a 20-fold excess of AEMA·HCl relative to dithiobenzoate groups was added. After purging of the reaction mixture for 20–30 min, the mixture of TEA and HexNH_2_ in DMF (0.1 M) was added by a syringe. The reaction mixture was stirred at room temperature for 24 h until it became a colorless solution. Then, solution was dialyzed against deionized water, concentrated and precipitated into acetone giving a white powder (yield 80 ± 5%). The modified polymer was analyzed by ^1^H NMR, UV–VIS and SEC. In ^1^H NMR spectra recorded in D_2_O at 60 °C the appearance of AEMA protons at 3.39 ppm (2H, -O-CH_2_-C*H*_2_-NH_2_) and 4.31 ppm (2H, -O-C*H*_2_-CH_2_-NH_2_) was detected.

### 2.6. Synthesis of Amphiphilic Hybrid Block-Copolymers

Amphiphilic block copolymers of poly(2-deoxy-2-methacrylamido-d-glucose)-*b*-poly(γ-benzyl-L-glutamate) (PMAG-*b*-PGlu(OBzl)) and poly(2-deoxy-2-methacrylamido-d-glucose)-*b*-poly(L-isoleucine) (PMAG-*b*-PIle) were obtained from step-wise ring-opening polymerization (ROP) of the appropriate amino acid *N*-carboxyanhydride (*M*) using PMAG-NH_2_ as a macroinitiator (macroI). All polymerizations were performed in Schlenk tubes under an argon atmosphere after deoxygenating the solution by three freeze–pump–thaw cycles. For polymerization a 2 wt% solution of macroI in DMF was applied. The [*M*]*/*[*macroI*] ratio ranged from 15 to 125. The reaction was carried out for 72 h at 30 °C under slight stirring. The product was precipitated in diethyl ether and then dialyzed using a membrane with MWCO 8000 against DMF, DMF/water and water to remove unreacted monomer and macroinitiator. The yields of block copolymers were in the range 42–59%. The composition of the PMAG-*b*-PGlu(OBzl) block copolymers was determined using ^1^H NMR spectroscopy by the comparing the relative integral areas of the 3 protons of the -CH_3_ groups of PMAG (0.6–1.5 ppm) to the five aromatic protons of Glu(OBzl) (7.27 ppm).

### 2.7. Polymer Characterization 

The structure and purity of polymers obtained from the syntheses outlined above were proved by ^1^H NMR at 25 and 60 °C in D_2_O, DMSO-d_6_, and CDCl_3_. The spectra were recorded using a Bruker AC-400 NMR spectrometer (400 MHz) (Karlsruhe, Germany). For the calibration of the chemical shift scale of the NMR spectra the corresponding solvent peak (4.78 ppm at 25 °C and 4.4 ppm at 60 °C (D_2_O), 2.52 ppm (DMSO-d_6_), 7.24 ppm (CDCl_3_) for ^1^H) was used. The characteristics of (co)polymers (molecular weights *M_n_* and *M_w_* and dispersity *Đ*) were determined by the use of size exclusion chromatography (SEC, Agilent-1260 Infinity, Santa-Clara, CA, USA) equipped with a triple-detection system: a dual angled laser light scattering, refractometric, and viscometric detectors. Then, the molecular weight characteristics of the samples of interest were determined by means of triple detection without calibration. The analysis was carried out at 50 °C using two Agilent PLgelMIXED-C columns (7.5 mm × 300 mm, 5 μm) and DMF with 0.1 M LiBr as eluent. The flow rate was set to 1.0 mL·min^−1^. 

The content of dithiobenzoate end groups in PMAG-CTA was determined by UV–Vis spectroscopy on a UV-1800 (Shimadzu, Kyoto, Japan) spectrometer (ε_305.5_ = 13,100 L mol^−1^cm^−1^; DMF). The content of CTA groups in polymer was calculated using Equation (3):

*ω* (*%*) = [*CTA groups*]/[*PMAG-CTA*] = (*D*_305.5_/*ε* × [*PMAG-CTA*]) × 100% (3)

where [*CTA groups*], [*PMAG-CTA*] correspond to the concentration of the initial and polymer CTA groups, respectively; *D_305.5_* corresponds to the optical density of PMAG-CTA at 305.5 nm; and *ε* is the extinction coefficient. 

The amount of amino groups in PMAG-NH_2_ was estimated by the TNBS method (ε_420_ = 1674 L·mol^−1^·cm^−1^; sodium borate buffer, pH 9.4). For plotting of the calibration curve the reaction of TNBS with AETL was utilized. The content of amino groups in the polymer was calculated by the use of Equation (4):

*ω* (*%*) = [*NH_2_ groups*]/[*PMAG-NH_2_*] = (*D*_420_/*ε* × [*PMAG-NH_2_*]) × 100% (4)

where [*NH*_2_ *groups*], [*PMAG-NH*_2_] correspond to the concentrations of NH_2_ groups and the polymer, respectively; and *D*_420_ is the optical density of PMAG-NH_2_ at 420 nm.

The content of isoleucine in the block copolymer was determined by HPLC amino acid analysis of hydrolyzed PMAG-*b*-PIle samples. The hydrolysis of 3 mg of a sample was carried out in 6 mL of 6 M HCl with 0.0001% phenol in a vacuum-sealed ampoule for four days. The solvent was evaporated several times by water to eliminate HCl and to reach finally the neutral pH value. The hydrolyzates were analyzed using a Shimadzu Liquid Chromatographic System (Kyoto, Japan) was equipped with two LC-20AD pumps, a SCL-10A VP system controller, an SPD-10AV scanning UV detector, a degasser, and a DGU-14A pump (Canby, OR, USA). The commercially available ultra-short monolithic columns, namely, CIM SO_3_ and CIM QA disks (3 mm × 12 mm i.d.), were applied as the stationary phases. Both monolithic disks were installed in one chromatographic cartridge (BIA Separations, Ajdovscina, Slovenia) and used in the conjoint mode of chromatography. The data was acquired and processed with LS Solution software (Shimadzu, Kyoto, Japan). UV detection was performed at 215 nm. Before analysis, the macromolecular fraction was removed under ultrafiltration through a membrane with MWCO 1000. For HPLC analysis, 0.02 M aqueous Na-acetic buffer, pH 4.0 (eluent A), 0.02 M Na-phosphate buffer, pH 7.0 (eluent B), and 0.02 M Na-phosphate buffer, pH 7.0, containing 1 M NaCl (eluent C) were used as components of the mobile phase. The separation was carried out at flow rate of 0.5 mL/min following the gradient program: 0–2 min—100% eluent A (pump A), 2–5 min—pump A purging and eluent change; 5–10 min—100% eluent B (pump A); 10–15 min—0–50% eluent C (pump B). The retention time for Ile was 7.2 min and for glucosamine 13.6 min. The amounts of glucosamine and isoleucine in the hydrolysate were calculated by the use of the preliminary built calibration curves for Ile and glucosamine, respectively.

The molecular weights and hydrodynamic radius *R*_h–D_ for macromolecules were measured by static and dynamic light scattering methods in solutions in DMSO at 21.0 °C. Light scattering was taken on a Photocor Complex unit (Photocor Instruments, Moscow, Russia); a Photocor-DL diode laser served as a light source (power of 5–30 mW, wavelength λ = 659.1 nm). The instrument was calibrated by benzene (*R_V_* = 2.32 × 10^–5^ cm^–1^). The correlation function of the scattered light intensity was obtained from the use of a Photocor-PC2 correlator with 288 channels and was processed by DynalS software. In these solutions, the asymmetry of light scattering was absent; thus, the *M*_w_ of the copolymers was determined by the Debay method. The refractive index increments were measured by a Refractometer RA-620 (KEM, Kyoto, Japan).

Thermogravimetric analysis (TGA) was carried out by the use of a Netzsch STA 449 F3 calorimeter (Selb, Germany) in a nitrogen atmosphere at a constant heating rate of 10 °C/min. The analysis was performed in the temperature range from 40 to 600 °C using the pre-homogenized samples.

### 2.8. Preparation and Characterization of Nanoparticles

The formation of polymer nanoparticles (NPs) was achieved by a phase inversion method (dialysis), as described elsewhere [43]. Self-assembled particles were then freeze-dried with the use of a LabConco lyophilic system (USA) and stored at 4 °C. Colloids of NPs were prepared by dispersing the dried particles for 60 seconds under sonication by means of the ultrasonic probe UP 50H Hielscher Ultrasonics (Teltow, Germany) in 0.01 M PBS at pH 7.4. The colloids were characterized by DLS measuring the hydrodynamic diameter, polydispersity index (PDI), and the ζ-potential of nanoobjects using a ZetasizerNano-ZS (Malvern, UK) equipped with a He–Ne laser at 633 nm at a scattering angle of 173° and 25 °C. 

The particle morphology was studied by scanning transmission electron microscopy (STEM) using a UHR FE-SEM SU8030 (Hitachi, Tokyo, Japan). Before analysis, a few drops of a colloid were placed onto a nickel grid covered by carbon and dried. Then, the grid was stained with 1% (*w*/*v*) uranyl acetate solution for 30–60 s and was used for the experiments after waiting for 24 h.

The scanning near-field optical microscopy experiments were carried out using a NeaSNOM microscope (Nea-SNOM, Martinsried, Germany) and SiC grids.

To study the stability of NPs, the colloids of polymer nanoparticles were prepared by dispersing 5 mg of the particles in 1 mL of PBS (0.01 M, pH 7.4) under sonication for 60 s. The solution was then tenfold diluted with DMEM and incubated for 24 h at 37 °C. The hydrodynamic particle diameter was determined by DLS. 

### 2.9. In Vitro Biodegradation Study

Before the biodegradation study all solutions were filtered through the sterile syringe PES membrane filters with pores of 0.45 μm (Membrane Solutions, Kent, WA, USA). The degradation process of PMAG-*b*-PIle particles was studied in two model media, namely, 0.02 M Clark-Labs buffer, pH 2.6, containing pepsin (1), and 0.01 M PBS, pH 7.4, with papain (2). Briefly, 0.5 mL of enzyme solutions (concentration 1 mg/mL) were introduced into 0.5 mL of suspension containing 1.0 mg of particles. These experiments were carried out at 37 °C. Every several days, a probe of 60 μL of supernatant was sampled for HPLC monitoring of Ile and glucosamine, as described in Section 2.7. To calculate the percentage of Ile and glucosamine, accumulated in the solution during biodegradation, the amount of these compounds formed during complete acidic hydrolysis of the copolymer was taken as 100%. 

### 2.10. Kinetics of Glutathione Sensitive Behavior of PMAG-b-PGlu(OBzl) 

The kinetics of glutathione-mediated NPs disruption, induced by reduction of disulfide bounds of PMAG-*b*-PGlu(OBzl), was investigated by DLS analysis. Reduced glutathione (GSH) was added to a 1 mg/mL suspension of NPs in PBS (0.01 M, pH 7.4) at a final concentration of 2 μM and 10 mM in a cuvette. The mixture was gently shaken at 37 °C. At predetermined time intervals the hydrodynamic diameter of the nanoparticles was measured. 

### 2.11. Paclitaxel Encapsulation 

1.0 mg of polymer was dissolved in 0.5 mL of DMSO and 100 μL of a solution of paclitaxel in DMSO with a concentration of 1.0 mg/mL was added. The mixture was ultrasonicated for 60 s, freeze dried, and the obtained formulation was redispersed under sonication for 60 s in the mixture of acetonitrile/0.01 M PBS (pH 7.4) = 1/10 (*v*/*v*). Free paclitaxel was removed via ultrafiltration with the use of tubes supplied with membranes (MWCO 3000, Amicon Ultra, Sigma-Aldrich). The filtrates were collected, lyophilized, and dissolved in 100 μL of acetonitrile. The final solution was analyzed using RP HPLC on ultra-short monolithic column C4 (3 mm × 12 mm i.d.) (BIA Separations, Ajdovscina, Slovenia) with UV-detection at 237 nm. Distilled water (eluent A) and acetonitrile (eluent B) were used as the mobile phases. The flow rate was adjusted to 0.5 mL/min. The injection volume was 20 µL. The HPLC analyses were carried out under gradient elution: 0–15 min–0–100% eluent B. The retention time for paclitaxel was 11.6 min.

The amount of encapsulated substances was determined from the difference between initial and non-encapsulated paclitaxel amounts. The loading capacity (LC) and encapsulation efficacy (EE) were calculated using following equations:

*LC* = (*m_i_* − *m_s_*)/*m_NP_* (5)

*EE* = (*m_i_* − *m_s_*)/*m_i_* × 100% (6)

where *m_i_* is the initial substance mass (µg), *m_s_* is the mass of non-encapsulated substance (µg), and *m_NP_* is mass of nanoparticles (µg).

### 2.12. Cell Culture Experiments 

To determine a cytotoxicity of empty NPs, HEK 293 and BEAS-2B cell lines were used. Paclitaxel-loaded particles were examined using A549 and MCF-7 cells in a concentration-depended manner. The concentration of empty nanoparticles was varied from 4 to 1000 µg/mL. The paclitaxel and drug-loading NP’s cytotoxicity was tested in a range of 0.4 to 200 ng/mL. HEK 293, BEAS-2B, A549 and MCF-7 cells were cultivated in a humidified environment at 37 °C/5% CO_2_. The medium was changed three times per week and the cells were subcultivated before reaching confluence using trypsin.

For cytotoxicity evaluation 8 × 10^3^ cells per well were seeded in a 96-well plate (100 μL/well) in the required culture medium and cultivated under a humidified atmosphere of 5% CO_2_ at 37 °C. After 24 h, the medium was replaced by the culture medium containing the test materials of different concentrations. Cell viability was determined after 24 or 72 h treatment using the CTB assay. The culture medium was removed and 100 μL of CTB working solution (10% stock solution in culture medium) were added to each well and incubated for 60–180 min. The number of viable cells was indirectly quantified by measuring the fluorescence intensity (λ_ex_ = 544, λ_em_ = 590 nm) using a microplate reader Fluoroskan Acent (ThermoFisher, Waltham, USA). For background correction the values measured for wells with CTB solution (without cells) were subtracted from those obtained from the presence of cells. The relative cell viability (%) was determined by comparing the fluorescence signals with control wells containing untreated cells. The data are presented as mean values ± SD (n = 4). 

The concentration-dependent normalized cell viability data obtained from CTB assays were fitted from 0 to 100 by using non-linear curve fitting/growth/sigmoidal/dose–response fitting functions (OriginPro 8.6). Half-maximal inhibition concentrations (IC_50_) were calculated from the fitted dose-response curves. 

## 3. Results and Discussion 

### 3.1. Synthesis of PMAG-CTA and Its Modification to Introduce Terminal Primary Amino Groups

In order to prepare the hybrid block copolymers, consisting of hydrophilic poly(2-deoxy-2-methacrylamido-D-glucose) (PMAG) and hydrophobic poly(L-isoleucine) or poly(γ-benzyl-L-glutamate), the sequential combination of two polymerization techniques was applied. The basic method for the synthesis of PMAG is radical polymerization, while poly(amino acids) are obtained from ring-opening polymerization of α-amino acid NCAs. The conjugation of water-soluble PMAG and hydrophobic poly(amino acids) represents a great challenge due to the impossibility to combine both polymers in the homogeneous reaction medium appropriate for conjugation. Mainly, it is related to the poor solubility (and sometimes total insolubility) of ordered hydrophobic poly(amino acids) even in organic media. Thus, the most appropriate strategy to prepare such amphiphilic copolymers includes: (1) synthesis of a glycopolymer PMAG-CTA via RAFT-polymerization; (2) post-modification of its terminal functional group to introduce a primary aliphatic amino group (PMAG-NH_2_); and (3) polymerization of α-amino acid NCA by the use of PMAG-NH_2_ as a macroinitiator (macroI).

The homopolymers PMAG-CTA were obtained via RAFT-polymerization of MAG in the presence of an initiator (AIBN) and a reversible chain transfer agent (CTA) as described earlier [41]. The characteristics of polymers obtained are presented in Table 1 (see ^1^H NMR spectrum in Supplementary Materials, Figure S1). 

When polymers are synthesized by RAFT polymerization, most of the polymer chains contain the active thiocarbonylthio-terminal groups (Z groups), which can be successfully functionalized. Among the different possible ways of modification of polymer Z groups, the most simple and convenient approach is the conversion of thiocarbonylthio groups into thiols with further introduction of the functionality of interest via different thiol-click reactions [44,45,46].

**Figure 1:**
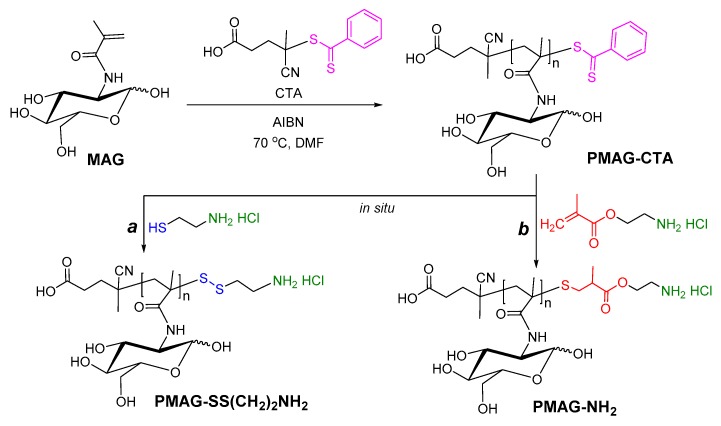
Scheme of RAFT-polymerization and modification of PMAG-CTA by: (**a**) 2-aminoethanethiol hydrochloride and (**b**) 2-aminoethyl methacrylate hydrochloride.

In present work, two strategies were utilized to modify thiocarbonylthio groups and to introduce the terminal amino functionality suitable for further ROP of NCAs: (1) the reduction of thiocarbonylthio groups into thiols followed by the reaction with 2-aminoethanethiol hydrochloride (AETL) (Figure 1a) [41]; and (2) the reduction of thiocarbonylthio groups into thiols followed by thiol-ene addition of 2-aminoethyl methacrylate hydrochloride (AEMA) (Figure 1b). In the first case, the modification of PMAG with AETL provides the formation of redox-responsive disulfide bonds, whereas PMAG-AEMA derivative contains a stable -S-CH_2_- linkage.

#### 3.1.1. Post-Modification of PMAG-CTA by 2-Aminoethanethiol Hydrochloride

Earlier, Wang et al. reported the development of an amino functionalization technique for RAFT-polymerized polystyrene. It is based on the one-pot process combining aminolysis of trithiocarbonate-terminal groups with an in situ thiol capping reaction by 2-(BOC-amino)ethyl methanethiosulfonate [47]. Deprotection of BOC groups allows for the release of primary amino groups to utilize them for further reactions.

In our case, it was suggested to obtain the amino functionalized PMAG from PMAG-CTA by the conversion of thiocarbonylthio groups into thiols followed by an in situ reaction of the polymer terminal thiols with AETL. To avoid the possible oxidation of polymer thiols and the side reaction of polymer dimerization, the modification was carried out in an argon atmosphere using a high excess of AETL in the reaction medium. As the nucleophilic agents, sodium borohydride [48] or the mixture of hexylamine with triethylamine [47,49] were applied to convert thiocarbonylthio groups into thiols. 

**Figure 2:**
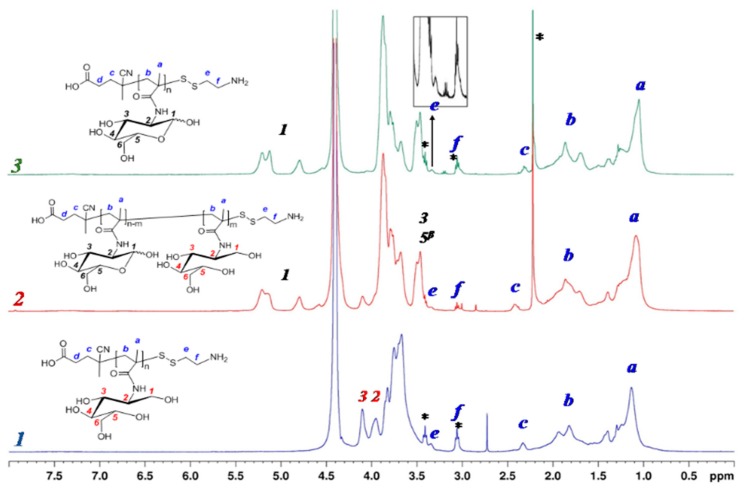
^1^H NMR spectra of PMAG modified by AETL: 1—NaBH_4_ in DMF/water (50/50, *v*/*v*%), 2—NaBH_4_ in DMF, 3—Et_3_N + HexNH_2_, DMF (* signals of low-molecular impurities—AETL or acetone).

The completeness of thiocarbonylthio groups modification was proved by disappearance of the absorption band with a maximum at 305.5 nm in the UV spectrum, which also was accompanied by the disappearance of pink color of the solution. Furthermore, the disappearance of the signal of aromatic protons at 7.4–7.9 ppm, corresponding to thiocarbonylthio groups in ^1^H NMR spectrum, also confirms polymer modification. In turn, the appearance of additional signals at 3.0 and 3.3 ppm relating to two CH_2_ groups of AETL gave evidence for the its successful attachment to the polymer chain (Figure 2).

At the same time, in the ^1^H NMR spectrum of PMAG-NH_2_ the change in signals of glycoside ring protons and the total disappearance of anomer protons in the MAG units was registered (Figure 2). It is known that in aqueous media the cyclic form of sugars can be converted into the opened one with the formation of an aldehyde group [50]. Since the process was performed in a DMF/water (50/50, *v*/*v*%) mixture, the results obtained can be related to the reduction of the aldehyde form of MAG units into hydroxyls in the presence of sodium borohydride. This assumption is also supported by the appearance of two signals in NMR spectra at 3.9 and 4.2 ppm, respectively, corresponding to protons at C^3^ and C^2^-atoms of alditol structure (Figure 2, spectrum 1 and Supplementary Materials, Figure S2, spectrum 1). 

To evaluate the effect of the reaction medium on the MAG structure, the modification of PMAG-CTA was also carried out in an organic medium (DMF) under the same conditions. In this case, the NMR signals, corresponding to both anomer protons and protons of glycoside ring of D-glucose, were observed in the ^1^H NMR spectrum of PMAG-SS(CH_2_)_2_NH_2_. Additionally, the signal at 4.2 ppm, corresponding to proton at C^2^, was also detected. However, the degree of reduced units (acyclic form) did not exceed 13% (Figure 2, spectrum 2 and Supplementary Materials, Figure S2, spectrum 2).

The alternative approach to convert Z groups of PMAG-CTA into thiols was the aminolysis using primary amines, such as hexylamine. In this case, the analysis of the with modified AETL polymer by ^1^H NMR-spectroscopy revealed the preservation of all proton signals of cyclic saccharide units of MAG (Figure 2, spectrum 3 and Supplementary Materials, Figure S2, spectrum 3). The characteristics of the initial PMAG-CTA and modified PMAG-NH_2_ homopolymers are presented in Table 2. Unlike the sample prepared via the modification with AETL in the presence of NaBH_4_ in a water-organic medium (sample 1), the other modification approaches revealed no dimerization during conversion of CTA groups into thiols (confirmed by SEC analysis, Supplementary Materials, Figure S3).

The content of amino groups in PMAG-NH_2_ was determined spectrophotometrically by the TNBS method [51]. For short PMAG samples (**#**1–4) the conversion of CTA groups calculated by this method agreed with the data determined by ^1^H NMR-spectroscopy. The low value of amino groups in long-chain PMAG (sample #5) determined spectrophotometrically can be related to the poor accessibility of terminal amino groups for interactions with TNBS. 

#### 3.1.2. Post-Modification of PMAG-CTA by 2-Aminoethyl Methacrylate Hydrochloride 

The thiol-ene addition was considered as an alternative route to introduce the terminal primary amino group into the polymer chain. This reaction can proceed either as a radical addition of thiols to unsaturated compounds under heat/light exposure or by means of base/nucleophilic catalyzed Michael addition of thiols onto an electron deficient ene-compound, such as acrylates, acrylamides, or maleimides [49,52]. In our case, AEMA was selected as the unsaturated compound containing the required primary amino group. The formation of thiols was fulfilled by aminolysis with HexA (in the presence of Et_3_N) in DMF. The reaction of resulting thiol groups with AEMA was carried out at room temperature for 24 h. 

**Figure 3:**
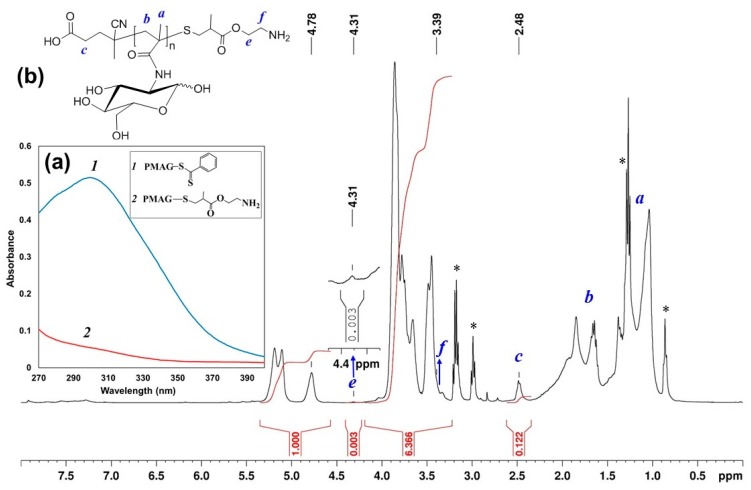
(**a**) UV spectrum of PMAG-CTA *(1)* and PMAG-NH_2_ obtained from modification by AEMA (*2*) in DMF; (**b**) ^1^H NMR spectrum of PMAG-NH_2_ (* low-molecular by-products Et_3_N and HexA).

The modified polymer was characterized by UV- and ^1^H NMR spectroscopy, as well as by SEC. Similar to modification by AETL, the disappearance of the absorption band with a maximum at 305.5 nm in the UV-spectrum was detected (Figure 3a), indicating the conversion of thiocarbonylthio groups. In the ^1^H NMR spectrum of PMAG-NH_2_, the signals of aromatic protons of the Z group disappeared, whereas the signals of both protons of CH_2_ groups of polymer-bound AEMA were found at 3.4 and 4.3 ppm, respectively (Figure 3b). According to SEC analysis, PMAG-NH_2_ was characterized by *M*_n_ = 5700 and *Ð* = 1.10 (Supplementary Materials, Figure S4). The degree of conversion according to ^1^H NMR-spectroscopy was found to be only 10%. Most probably, such low modification efficiency is related to the non-optimum reaction conditions (22 °C, 24 h).

According to the literature, the efficient modification of the RAFT-agent by aminolysis followed by Michael thiol-ene addition can be achieved at elevated temperature (70 °C) for 48 h [53]. Taking this into account we performed the modification of PMAG-CTA by AEMA in an inert atmosphere at 70 °C. In this case, the content of amino groups of PMAG-NH_2_ determined by the TNBS method was much lower than the amount of AEMA detected by ^1^H NMR-spectroscopy (Table 2, sample 7). The molecular weight of modified polymer determined by SEC using triple detection was found to be 53,000. Both facts indicate evidence for crosslinking of the polymer that most probably can be related to the reaction of acyclic glucose units with the amino groups of AEMA. Thus, the modification of PMAG by AETL, both using NaBH_4_ and Et_3_N + HexA, was more successful than the derivatization of PMAG by AEMA.

### 3.2. Synthesis and Characterization of Hybrid Block Copolymers of PMAG-b-P(Amino Acid) 

**Figure 4:**
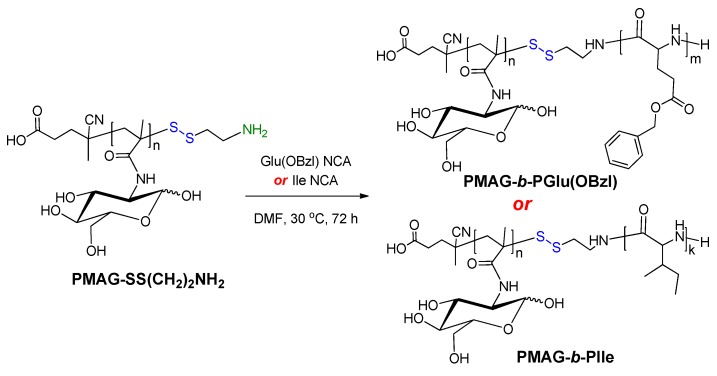
Scheme of synthesis of amphiphilic PMAG-*b*-P(amino acid).

PMAG-NH_2_ obtained via modification with AETL, was applied as a macroinitiator for the polymerization of NCA of hydrophobic α-amino acids L-isoleucine (Ile) and γ-benzyl-L-glutamate (Glu(OBzl)). The scheme of copolymerization is presented in Figure 4. As poly-L-isoleucine, poly(γ-benzyl-L-glutamate) is also biodegradable [54]. Moreover, the partial removal of the protective Bzl groups allows for the modification of released carboxyl groups of the polymer, for example, with the aim of covalent attachment of a drug or fluorescent dye. 

**Figure 5:**
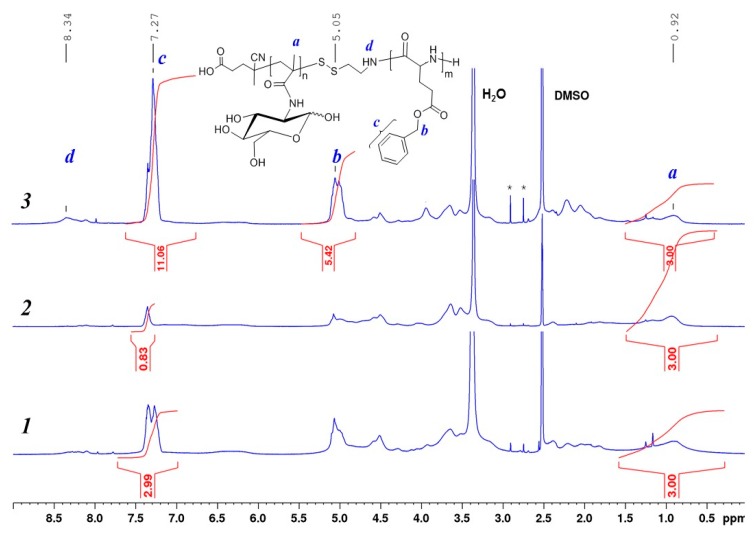
^1^H NMR spectra (DMSO-d_6_, 25 °C) of block copolymers PMAG-*b*-P(Glu(OBzl)) with different composition (Table 2): *1*—sample *b*4, *2*—sample *b*1, *3—*sample *b*6.

The series of PMAG-*b*-P(amino acid) with different length of the hydrophilic and hydrophobic blocks was obtained and the characteristics of copolymers are summarized in Table 3. The structure and the composition of PMAG-*b*-PGlu(OBzl) was confirmed by ^1^H NMR- and IR-spectroscopy, as well as by SEC analysis. ^1^H NMR spectra of PMAG-*b*-PGlu(OBzl) with different composition of MAG and Glu(OBzl) units are presented in Figure 5. As can be seen, the signals at 5.03 and 7.27 ppm, corresponding to CH_2_ and aromatic protons of the Bzl group of Glu(OBzl) monomer units, are observed. The copolymer composition was determined from the ratio of relative integral areas of three protons of the CH_3_ groups of PMAG observed at 0.7–1.5 ppm to the five protons of the aromatic ring at 7.27 ppm belonging to the PGlu(OBzl) block.

**Figure 6:**
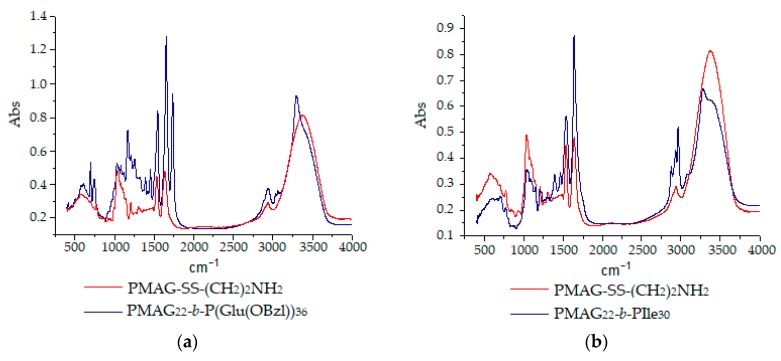
IR-spectra of homopolymer PMAG-NH_2_ and PMAG-*b*-P(amino acid) block-copolymers: PMAG-*b*-PGlu(OBzl) (**a**) and PMAG-*b*-PIle (**b**).

Additionally, in comparison with PMAG-NH_2_, in the IR-spectrum of PMAG-*b*-PGlu(OBzl), the bands of the aromatic ring at 1453, 3035, and 3065 cm^−1^ and the ester group at 1168, 1256, and 1735 cm^−1^ were detected (Figure 6a). SEC analysis with triple detection for PMAG-*b*-PGlu(OBzl) samples demonstrated a non-adequate relation between the copolymer composition and the molecular weight, whereas static light scattering (SLS) revealed a regular increase in molecular weight of amphiphilic copolymers with the growth of the hydrophobic block length (Table 3). The details on the static and dynamic light scattering analysis of the polymers can be found in the Supplementary Materials (Table S1).

Contrary to PMAG-*b*-PGlu(OBzl), PIle-containing copolymers showed poor solubility due to the formation of a secondary structure caused by poly-L-isoleucine. In particular, poly-L-isoleucine has the ability to form β-sheet structures, which are known to have a poorer solubility than α-helixes or disordered coils [55,56]. The tendency to form a colloid was observed even in organic solvents. For this reason, to confirm the structure and determine the composition for PMAG-*b*-PIle, IR-spectroscopy and HPLC analysis were applied. In accordance with Figure 6b, the characteristic bands at 1389, 1463, 2879, and 2966 cm^−1^ were recorded for PMAG-*b*-PIle. The detected bands corresponded to the valence asymmetric, valence symmetric, deformation asymmetric, and symmetric vibrations of -CH_3_ groups. The composition of PMAG-*b*-PIle was determined using quantitative HPLC analysis of isoleucine and *N*-glucosamine after acidic hydrolysis of copolymers (Table 3).

The analysis of PMAG-*b*-PIle samples by SEC in DMF with 0.01 M LiBr (60 °C) with triple detection gave inflated values of molecular weight which can be attributed to the formation of aggregates. The determination of molecular weight by static light scattering (SLS) in DMSO allowed for the detection of single macromolecules with a molecular weight of 7700. The molecular weight of 7700 determined by SLS appeared to be closer to the value calculated from the found polymer composition (9075). With the use of DLS analysis it was found that the hydrodynamic diameter of macromolecules was ≤ 1 nm. In comparison with other copolymers of comparable or lower molecular weight (samples *b1* and *b2*, Table S1 of Supplementary Materials), the determined hydrodynamic diameter indicates a collapsed conformation that may be a result of the formation of ordered β-sheet structures in the polypeptide block. 

**Figure 7:**
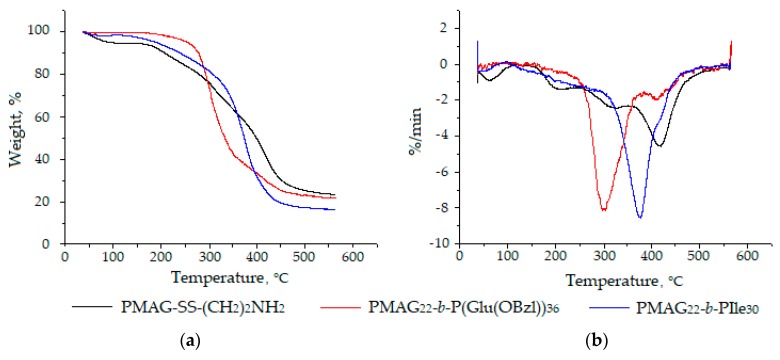
Thermogram *(***a***)* and differential thermogram *(***b***)* of PMAG-SS(CH_2_)_2_NH_2_ homopolymer and its copolymers with poly(amino acids).

Additionally, a thermogravimetric analysis (TGA) was performed for both types of block-copolymers. TGA of macromolecules is a tool to determine the thermal stability of polymers, as well as to compare their stability to that of homopolymers. It is known that glycopolymers are highly hygroscopic and characterized by an initial mass loss up to 160 °C, associated with evaporation of adsorbed water [57]. The destruction of the homopolymer PMAG-SS(CH_2_)_2_NH_2_, as many other glycopolymers [57,58], proceeds in three stages (Figure 7). In the first and second stages, the carbohydrate side chain is decomposed to water and carbon dioxide, followed by the destruction of the residual macromolecular chain [58]. 

According to the literature, thermal degradation of polypeptides is known to occur in the temperature range between 150–450 °C and represents an one-step process [59]. In our case, the degradation profiles of copolymers also have fallen in this known temperature interval (Figure 7). The 5% mass loss was observed at 189 °C for PMAG-*b*-PIle (sample *b6*) and at 226 °C for PMAG-*b*-PGlu(OBzl) (sample *b4*), respectively. A 50% mass loss of the same samples was detected at 374 and 334 °C, respectively. The most intense decomposition of copolymers was observed at 300 °C for PMAG-*b*-PGlu(OBzl) and 380 °C for PMAG-*b*-PIle (Figure 7b), respectively.

### 3.3. Preparation and Characterization of Self-Assembled Nanoparticles

The preparation of polymer nanoparticles (NPs) based on synthesized block copolymers was carried out due to self-assembly during solvent inversion (dialysis) followed with freeze-drying for storage. Before use, the polymer samples were redispersed in water or 0.01 M PBS via short-term ultrasonic exposure (30 s). Self-assembly of block copolymers occurs due to hydrophobic interactions between hydrophobic regions of macromolecules in organic-aqueous or aqueous media. Hydrophobic fragments of macromolecules were assembled to reduce the area of contact with water, whereas the hydrophilic parts were exposed into aqueous medium because of their high solubility in water. For self-assembly of block copolymers one can expect the formation of following structures: spherical and cylindrical micelles as well as polymerosomes [60]. The formation of well-defined structures depends both on the chemical nature of the block-copolymer and the relative length of the hydrophobic and hydrophilic fragments.

The diameter of the NPs was analyzed using dynamic light scattering (DLS), scanning transmission electron microscopy (STEM), and scanning near-field optical microscopy (SNOM). Contrary to DLS, where the hydrodynamic diameter is measured, both STEM and SNOM analysis allow one for determining the diameter of dry NPs. The values of hydrodynamic diameter (*D_H_*) of all NPs measured in 0.01 M PBS (pH 7.4) seemed to be close to each other and varied from 180 to 260 nm (Table 4). 

**Figure 8:**
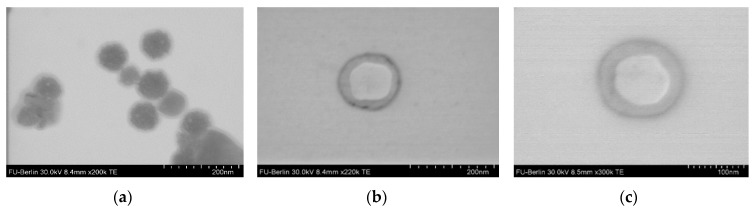
STEM images of nanoparticles based on (**a**) PMAG-*b*-PGlu(OBzl) (sample *b5*) and (**b**,**c**) PMAG-*b*-PIle (samples *b7* and *b8*).

Moreover, the analysis of NPs by STEM allowed us to reveal of a difference between NPs based on PMAG-*b*-PGlu(OBzl) and PMAG-*b*-PIle (Figure 8, Figure S5 of Supplementary Materials). 

In the case of PMAG-b-PGlu(OBzl), a drastic difference compared to D_H_ was observed: the diameter detected by STEM was 3-4 times smaller than D_H_. In turn, the diameter of PMAG-b-PIle NPs determined by DLS and STEM differed only twice. These characteristic differences in size hint changes in morphology of NPs based on PIle- and Glu(OBzl)-containing copolymers. It is known that diblock-copolymers can self-assemble into star-like or large compound micelles, or polymersomes [6,61]. According to STEM, PMAG-b-P(Glu(OBzl)) was found to be self-assembled into micelles with a hydrophobic core (dark in the TEM image) and a hydrophilic corona (light grey area around dark core in the TEM image) (Figure 8a). It is known that polymer star-like micelles are similar to surfactant micelles and their diameter is about 30–50 nm [61]. Large compound micelles are quite large (up to 1200 nm) and possess self-segregated domains. Since the formed nanoparticles are quite small, most probably they are star-like micelles. In turn, PMAG-b-PIle formed polymersomes that have a hydrophilic core (light grey core in TEM) and a hydrophobic membrane (dark ring around the light grey core in TEM) (Figure 8b,c) with a thickness of 20 ± 5 nm (Figure 8b,c). Most probably the difference in solubility of copolymers and morphology of the NPs under study is related to the formation of a β-sheets in the case of PIle [55,56] and α-helixes in the case of PGlu(OBzl) [62].

### 3.4. In Vitro Biodegradation Study

Investigations on the biodegradation of the polymer particles is a necessary step in the development of materials based on biodegradable polymers. Usually, in vitro biodegradation is carried out in model media containing enzymes and yields important information on the rate of polymer degradation. Obviously, an introduction of non-degradable fragments into the structure of biodegradable systems can change the kinetics of polymer particles’ biodegradation. 

The developed PMAG-*b*-poly(amino acid) NPs consist of two fragments of different biodegradability. The hydrophobic block based on poly(amino acid) is completely biodegradable. In this case, the process of destruction occurred due to the action of various proteases. As to PMAG, its backbone is non-biodegradable, whereas the cleavage of glucosamine of the side chain seems to be possible. If that happens, PMAG will turn into poly(methacrylic acid), which is also a water-soluble polymer. Since the molecular weights of the used PMAG samples do not exceed 30,000, the water-soluble polymer remaining after poly(amino acid) degradation can be excreted from the body via kidney filtration. 

To evaluate the ability to biodegradation, PMAG-*b*-PIle (sample *b7*) NPs were used. Thiol proteinase of plant origin papain and the digestive enzyme pepsin were used as model biocatalysts. Papain, which acts at physiological conditions, is able to catalyze the hydrolysis of peptide bonds as well as ester and amide bonds formed by amino acids. Pepsin is an endoprotease, which acts at acidic pH (with a maximum at pH 2.0) with proteolytic cleavage of the bonds formed between different α-amino acids. 

**Figure 9:**
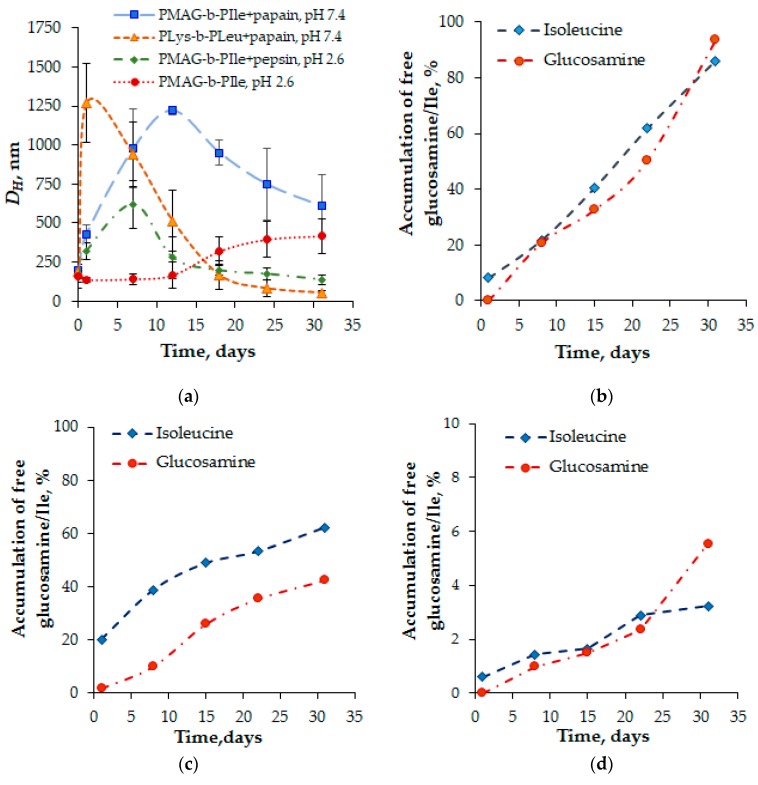
Monitoring of the biodegradation process of (PMAG-*b*-Ile)-based particles (sample *b7*) at 37 °C in different media: (**a**) changes in hydrodynamic diameters of PMAG-*b*-Ile and PLys-*b*-PLeu NPs (DLS); (**b**–**d**) accumulation of free isoleucine and glucosamine (HPLC) in: 0.02 M Clark-Labs buffer, pH 2.6, containing pepsin (**b**); 0.01 PBS, pH 7.4, containing papain (**c**), and human blood plasma (**d**).

The degradation process was monitored using two approaches: (1) by measuring the change in *D_H_* of particles during the biodegradation process by DLS; and (2) by determining the free amino acids and glucosamine accumulation in the reaction medium using HPLC. As a benchmark, the totally biodegradable block copolymer of L-lysine and L-leucine containing approximately 100 units of each amino acid was used for this study. Figure 9a illustrates the change in hydrodynamic diameter of the nanoparticles within a month. As it was expected, the highest and fastest aggregation of particles was observed for PLys-*b*-PLeu-based nanomaterials. Such a dramatic increase in size can be associated with the fast degradation of the hydrophilic block (PLys), which stabilized the inner hydrophobic core in an aqueous medium. In turn, it led to the reorganization of the polymer due to hydrophobic interactions and, as a result, to aggregation of NPs. At the next stage, the degradation of shorter hydrophilic fragments and gradual depolymerization of the hydrophobic polymer took place. In a month, the hydrodynamic diameter of such particles was reduced from 190 to 50 nm. 

In the case of hybrid copolymers, considerable aggregation of the particles based on PMAG-*b*-PIle was observed after co-incubation with an enzyme within one week for the pepsin-containing system. At the same time, the control system, representing NPs incubated just in a buffer at pH 2.6 without enzymes, was stable for at least 12 days. This means that the increase in *D_H_* of the NPs incubated in enzyme-containing media is evidently related to the polymer biodestruction. In the case of papain, the aggregation process reached its maximum after two weeks. In both cases, a time-dependent decrease in particle size was observed, but for the system containing pepsin it was faster. 

This finding is also supported by HPLC data. In particular, it was established that the accumulation of free glucosamine and isoleucine in an acidic medium occurred much faster than at pH 7.4 (Figure 9b,c). Moreover, it is noted that at acidic pH, the accumulation of glucosamine and isoleucine was found to be comparable, whereas, at physiological pH, the degradation of isoleucine occurred faster. We speculate that, at physiological pH, the process is specific and mostly controlled by enzymatic action. In turn, at acidic pH the degradation mechanism seems to be mixed and combines not only the enzymatically catalyzed process, but also an additional acidic hydrolysis of the amide bond.

Additionally, the experiment on biodegradation of PMAG-*b*-PIle NPs was carried out in human blood plasma at 37 °C (Figure 9d). In this case, the degradation rate was significantly lower and the content of low-molecular weight products did not exceed 6% after one month of incubation. For a comparison, the degradation of poly(L-amino acid)-based nanoparticles, e.g., PLys-*b-*PLeu, in plasma for the same time period resulted in the detachment of 28% of Lys and 17% of Leu. It is important that both PLys-*b-*PLeu and PMAG-*b*-PIle had identical particle morphology, namely, they were polymersomes. Thus, NPs based on developed hybrid polymers are more resistant to biodegradation as compared to NPs based on natural amino acids. 

### 3.5. Redox-Responsive Studies 

Drug delivery systems can be tuned to release drugs by changes in the microenvironment, such as temperature, tumor pH, enzyme activity, or the presence of redox agents or the redox state of the biological environment. Polymer NPs containing disulfide bonds have the unique property via cleavage of this bond that allows nanocarriers to degrade and release loaded cargoes in response to the presence to a reducing environment of the cell [63]. 

Glutathione (GSH) is the most common reducing agent in living cells. It is known that the intracellular GSH concentration (10 mM) is significantly higher than the level in the cellular exterior (2 μM). Therefore, the delivery systems with the GSH-mediated release the entrapped drugs in response of the glutathione level. They can be stable in the extracellular space and can be destructed inside cells. The facilitated release of encapsulated drugs at the cell cytosol can be achieved by the disruption of polymer particles, containing disulfide bonds. During this process GSH reduces S–S bonds linking hydrophobic and hydrophilic blocks in copolymers and is converted to its oxidized form, glutathione disulfide (GSSG). 

**Figure 10:**
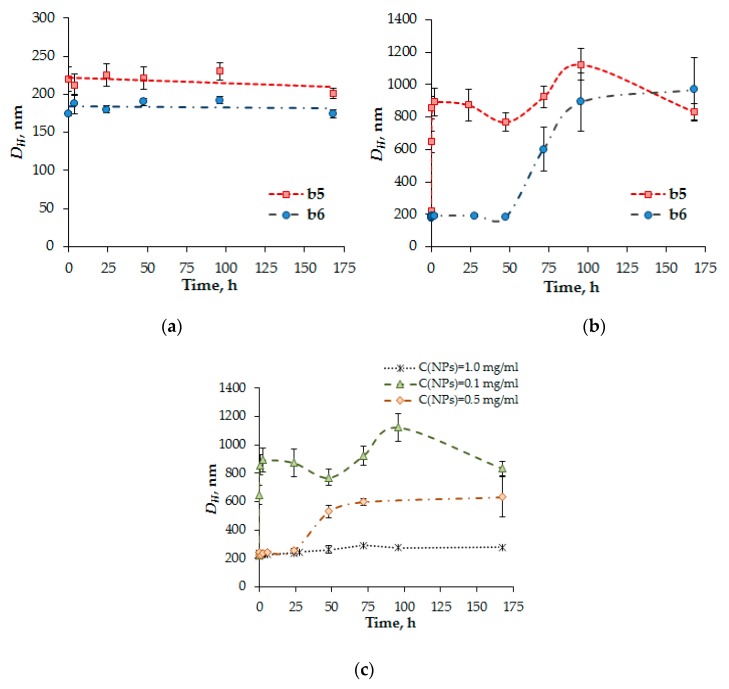
The kinetics of the glutathione-mediated NPs disruption induced by reduction of disulfide bonds of PMAG*-b*-PGlu(OBzl). Reaction conditions: (**a**) C_NPs_ = 0.1 mg/mL, C_GSH_ = 2 µM; (**b**) C_NPs_ = 0.1 mg/mL, C_GSH_ = 10 mM; (**c**) C_GSH_ = 10 mM.

The kinetics of glutathione-mediated NPs disruption, induced by the reduction of disulfide bonds in PMAG-*b*-PGlu(OBzl), was investigated by DLS. Figure 10 shows the change in *D_H_* of the particles as a function of time in the presence of GSH at different concentrations.

At extracellular GSH level (2 μM) the particles were stable and did not change their diameter *D_H_* (Figure 10a). In turn, the particles at intracellular GSH concentration (10 mM) were disrupted and aggregated immediately after adding GSH (Figure 10b). It is worth noting that increasing the length of the hydrophilic PMAG block in the copolymer from 22 to 80 monomer units increases the stability of polymer NPs with respect to disruption. Thus, the variation of hydrophilic block length allows the control of the rate of the glutathione-mediated NPs’ disruption and, consequently, the rate of release encapsulated drugs. 

The interaction of particles with glutathione was also studied at different concentrations of particles (Figure 10c). In this case, an increase in NP concentration from 0.1 to 1.0 mg/mL followed with an extension of the polymer NPs’ life-time, which is associated with an increase in the concentration of disulfide bonds. Thus, it can be assumed that at the initial stage of accumulation of NPs in cells the rapid destruction of particles and, therefore, rapid drug release will occur. A further increase in concentration of NPs inside cells might favor to slow the destruction process down due to the change in ratio of NPs/GSH.

### 3.6. Cytotoxicity Study 

**Figure 11:**
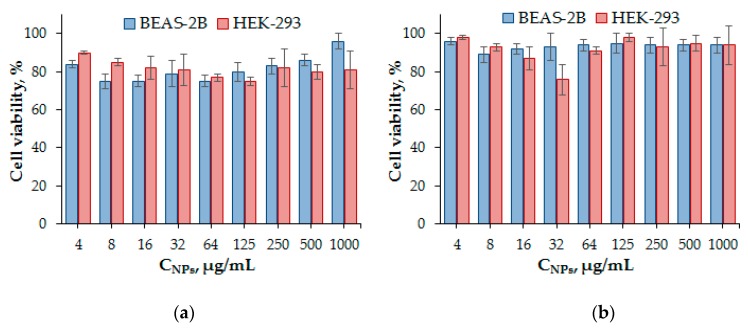
Viability of human embryonic kidney (HEK 293) and human lung cells (BEAS-2B) after their incubation for 3 days in the presence of PMAG-*b*-PGlu(OBzl) micelles (sample *b5*) (**a**) and PMAG-*b*-PIle polymersomes (sample *b7*) (**b**).

In vitro cytotoxicity of NPs was studied using human embryonic kidney (HEK 293) and human lung cell lines (BEAS-2B) in order to evaluate their cytotoxic profile. The cell viability was determined after 24 or 72 h treatment, respectively, using the CTB Assay that is based on the reduction of blue resazurin to purple resorufin by metabolically-active cells. The results showed the absence of cytotoxicity of both types of PNPs at concentrations up to 1.0 mg/mL (Figure 11) and, thus, demonstrated the great potency of these materials for use as drug delivery systems.

In the current literature, it is broadly indicated that the particle size is one of the key factors influencing the in vivo behavior of NPs [1]. Moreover, the effect of particle charge on the stability in the bloodstream has also been discussed [64]. However, the dominant part of papers is limited to study the particle stability only at physiological pH in buffer or saline solutions [65] while their behavior in model media containing proteins, which can have a strong effect on particle size and stability via aggregation, is hardly discussed.

**Figure 12:**
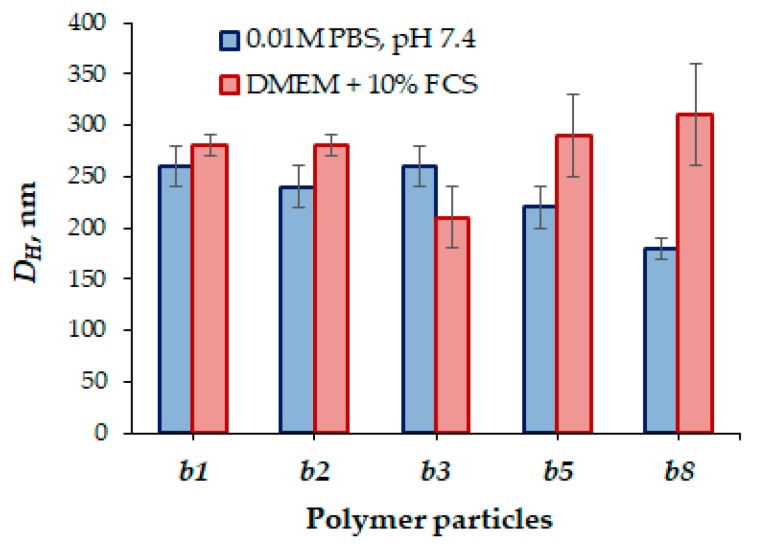
Hydrodynamic diameters of nanoparticles based on PMAG-*b*-PAA of different compositions in the buffer (0.01 M PBS, pH 7.4) and culture (DMEM + 10% FCS) media.

In this work, cell culture media containing 10% of fetal calf serum (FCS) was chosen as a model medium. The hydrodynamic diameter based on the PMAG-*b*-PAA hybrid copolymers did not change significantly and the particles remained stable with respect to aggregation (Figure 12). Nevertheless, with the elongation of the hydrophobic block (samples *b5* and *b8*), a slight tendency toward an increase in particle size in the presence of proteins was observed.

### 3.7. Paclitaxel Delivery Systems

**Figure 13:**
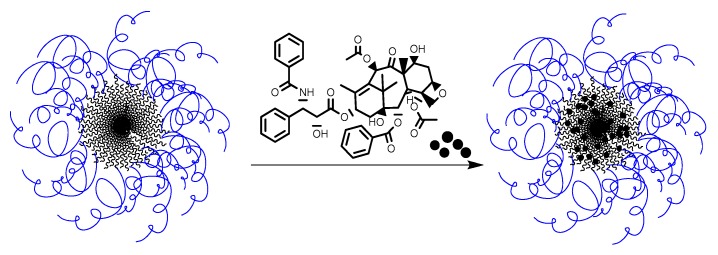
Scheme of paclitaxel encapsulation into PMAG-*b*-PGlu(OBzl) micelles.

With the aim to increase the solubility and bioavailability of paclitaxel (PTX) for improving drug safety, prolonging the circulation half-life, and to improve the efficacy and reduction of side effects, new formulations of paclitaxel based on the developed PMAG-*b*-PAA NPs were studied (Figure 13). 

Initially, PTX was encapsulated into the particles in a mixture of water and an organic medium (acetonitrile:water = 1:10) which was then replaced by water during dialysis. In this case, the maximum achieved encapsulation efficiency was 25%. The low encapsulation efficiency of PTX into the particles, may be associated with crystallization of the hydrophobic drug in the external aqueous phase when the organic solvent was removed during the dialysis [66]. 

To increase the efficiency of drug loading, as well as to avoid crystallization of PTX in the solution, another method for drug encapsulation into polymer particles was proposed. The encapsulation was carried out as follows: the copolymer and the drug were dissolved in DMSO, the mixture was lyophilized, and then redispersed in 0.01 M PBS, pH 7.4, under sonication with the subsequent removal of the unbound drug by dialysis against the water-organic medium (acetonitrile:water = 1:10). This approach allowed for an encapsulation of >90% of the drug (90 μg/mg of NPs).

Despite the similar encapsulation efficiency for both micelles (PMAG-*b*-PGlu(OBzl) and polymersomes (PMAG-*b*-PIle), the localization of the drug inside the particles was different. Being hydrophobic PTX localizes inside the hydrophobic core of polymer micelles or the hydrophobic membrane of polymersomes. Therefore, the encapsulation of the hydrophobic drug can influence the hydrodynamic diameter of polymer NPs. When paclitaxel was encapsulated into polymer micelles their sizes remained stable if the hydrophilic/hydrophobic ratio was large enough (Table 5, samples *b1–b3*, *b6*). In turn, the particle size was significantly increased, if the hydrophobic block contains a long chain and the hydrophilic corona could not stabilize the swollen core due to the encapsulated PTX (Table 5, sample *b5*). Additionally, the encapsulation of PTX into polymersomes led to a drastic increase of the NPs’ hydrodynamic diameter (Table 5, samples *b7*, *b8*).

The in vitro anti-tumoral activity of paclitaxel-loaded particles on A549 (human lung carcinoma cells) and MCF-7 (human breast adenocarcinoma) cancer cells was evaluated (Table 5). As a benchmark, the commercially-available formulation Paclitaxel-LANS^®^ was used. In the case of A549 cells, *IC*_50_ values determined for developed encapsulated forms of drug were very close to Paclitaxel-LANS^®^. In turn, the activity of encapsulated forms against MCF-7 cells were higher than for commercial formulation. In spite of this fact, the encapsulated forms of paclitaxel may reduce side effects under systemic administration. In general, the present results demonstrate the high potency of the developed NPs as drug delivery system for the hydrophobic anti-tumor drug paclitaxel. 

## 4. Conclusions

In this research two different approaches were applied for the preparation of macroinitiators based on the glycopolymer poly-2-deoxy-2-methacrylamido-D-glucose (PMAG) for ROP of α-amino acid NCAs. The first one included the transformation of dithiobenzoate end groups to thiols by the treatment with sodium borohydride or hexylamine with triethylamine followed by an in situ reaction of the -SH groups with AETL. This scheme allowed for performing both reactions with high yields and, most importantly, the synthesis of polymers containing glutathione-sensitive disulfide bonds. The second route was based on the application of hexylamine as a nucleophilic agent for introducing thiol groups and their further thiol-ene addition to 2-aminoethyl methacrylate. However, in this case only a low degree of conversion of CTA groups was achieved without the undesirable side reactions.

PMAG of different length, containing primary NH_2_ groups, was used for the ROP of Glu(OBzl) and Ile NCAs and the amphiphilic block copolymers with a hydrophilic non-biodegradable PMAG block and biodegradable hydrophobic poly(amino acid) block were obtained and applied for particle preparation. According to DLS the hydrodynamic diameter of the nanoparticles prepared at optimum conditions was around 200 nm. The morphology of PMAG-*b*-Ile particles evaluated by TEM can be identified as polymersome-like, whereas PMAG-*b*-PGlu(OBzl) particles represented micelles. 

The degradation process of the particles was monitored using two model enzymatic systems and human blood plasma. PMAG-containing nanoparticles proved to be more stable against enzymatic degradation than those based only on poly(amino acids). The NPs obtained did not induce cell death up to the concentration 1.0 mg/mL and possessed glutathione-mediated NPs disruption that could vary depending on hydrophilic block length. 

In order to increase the solubility and bioavailability of paclitaxel, new formulations of this drug were developed. The in vitro anti-tumoral activity of paclitaxel-loaded particles on A549 (human lung carcinoma cells) and MCF-7 (human breast adenocarcinoma) cells was evaluated. The use of encapsulated forms of the drug allowed to achieve a comparable efficacy as commercially available Paclitaxel-LANS. Thus, the present results demonstrate the great potency of the developed NPs with prolonged stability and glutathione-mediated disruption as a drug delivery system that appears to be useful for hydrophobic anti-tumor drugs.

## Figures and Tables

**Table 1:** RAFT-polymerization of 2-deoxy-2-methacrylamido-D-glucose: Characteristics of homopolymers depending on the synthesis conditions.

Sample #	Reaction Conditions	PMAG-CTA Characteristics					
	[MAG]_o_:[CTA]_o_:[AIBN]_o_	*ω_MAG_, %*	*x ^a^,* *%*	*M_n_^th (b)^*	SEC		
					*M*_n_	*M*_w_	*Ð*
1	20:1:0.25	10	~70	3700	4600	4800	1.05
2	20:1:0.25	20	~76	4000	6100	6400	1.05
3	75:1:0.25	20	~75	14,100	20,300	21,700	1.07

*^a^* Conversion was determined by ^1^H NMR spectroscopy in D_2_O; *^b^* Calculated using the Equation (2).

**Table 2:** Characteristics of the initial PMAG-CTA and modified PMAG-NH_2_ homopolymers.

Sample #	Thiol Generation Technique	Initial Polymer PMAG-CTA			Modified Polymer PMAG-NH_2_			
		(CTA Groups), %	*M*_n_ *^a^*	*Ð*	(NH_2_ Groups), %	Degree of Conversion, % (TNBS/NMR *^b^*)	*M*_n_ *^a^*	*Ð*
**Modification by AETL**								
1 *^c^*	NaBH_4_	3.2	4600	1.03	2.20	70/57	8700	1.67
2	NaBH_4_	2.1	4600	1.05	1.70	80/58	5400	1.05
3	NaBH_4_	2.2	6100	1.05	1.00	45/37	6000	1.04
4	Et_3_N+HexA	2.2	6100	1.05	1.60	73/55	6500	1.08
5	NaBH_4_	1.1	20,300	1.07	0.25	23/60	23,400	1.04
**Modification by AEMA**								
6	Et_3_N+HexA (22 °C)	2.1	4600	1.05	1.70	83/10	5700	1.10
7	Et_3_N+HexA (70 °C)	2.1	4600	1.05	0.80	36/~80 ^d^	53,000	1.17

*^a^* determined by SEC in triple detection; *^b^* end-group functionality calculated by ^1^H NMR according to the ratio of the integral proton intensities of the polymer CH_2_- R-group (c) CTA, with respect to CH_2_-group AETL (or AEMA) (e, Figure 2 and Figure 3); *^c^* reaction was carried out in water-organic medium; *^d^ an* approximate value due to poor resolution of signals in ^1^H NMR spectrum.

**Table 3:** The characteristics of synthesized amphiphilic hybrid copolymers.

Sample #	PMAG-NH_2_ (*macroI*)			Block Copolymer Characterization				
	*M*_w_*^a^* (SEC)	*Ð*	*M*_w_*^b^* (SLS)	Approx. Copolymer Composition		*M*_w_*^a^* (SEC)	*Ð*	*M*_w_*^b^* (SLS)
				MAG Units *^c^*	Glu(OBzl) *^c^* or Ile *^d^* Units			
PMAG-*b*-P(Glu(OBzl)								
*b*1	5600	1.05	4200	22	4	7000	1.07	5700
*b*2				22	9	7400	1.30	9000
*b*3				22	15	11,200	1.35	10,700
*b*4				22	48	11,500	1.45	
*b*5				22	60	11,700	1.35	21,400
*b*6	24,300	1.04	18,500	80	48	29,600	1.27	25,000
PMAG-*b*-PIle								
*b*7	5600	1.05	4200	22	30	28,200	1.05	7700
*b*8				22	38	33,400	1.07	

*^a^* SEC with triple detection was performed in DMF with 0.01 M LiBr, 60 °C; *^b^* Static light-scattering was carried out in DMSO; *^c^* determined by ^1^H NMR-spectroscopy; *^d^* determined by quantitative amino acid HPLC analysis.

**Table 4:** Physico-chemical characteristics of self-assembled NPs (in water).

Block-Copolymer	DLS			STEM	SNOM
	ζ-Potential, mV	*D_H_^a^,* nm	PDI	*D ^b^,* nm	*D ^b^,* nm
*b1*	−6.1 ± 0.4	260 ± 20	0.22 ± 0.06		
*b2*	−6.4 ± 0.1	240 ± 20	0.22 ± 0.06		
*b3*	−10.4 ± 0.3	260 ± 20	0.18 ± 0.03		
*b5*	−13 ± 2	220 ± 20	0.17 ± 0.02	50 ± 30	
*b6*	−8 ± 2	180 ± 10	0.24 ± 0.04		
*b7*	−17± 2	200 ± 40	0.21 ± 0.05	100 ± 50	130 ± 60
*b8*	−15± 2	260 ± 60	0.30 ± 0.06	150 ± 70	130 ± 50

*^a^* Hydrodynamic diameter of the particles calculated as mean value for three measurements of three separate suspensions; *^b^* Diameter of the particles calculated as the mean value of 50 objects.

**Table 5:** Characteristics of empty and paclitaxel-loaded PMAG-*b*-PAA NPs and cytotoxic effect of encapsulated forms of paclitaxel.

Sample #	*D_H_* of NPs, nm		*IC*_50_, ng/mL	
	Empty	Loaded	A549 Cells	MCF-7 Cells
PTX-LANS^®^			2.0 ± 0.1	4 ± 1
*b1*	260 ± 20	240 ± 30	5.5 ± 0.4	45 ± 6
*b2*	240 ± 20	190 ± 10	1.8 ± 0.1	8 ± 2
*b3*	260 ± 20	300 ± 40	2.4 ± 0.1	40 ± 10
*b5*	220 ± 20	710 ± 40	2.1 ± 0.2	13 ± 2
*b6*	180 ± 10	200 ± 10	2.9 ± 0.3	23 ± 2
*b7*	200 ± 40	890 ± 40	3.7 ± 0.1	12 ± 2
*b8*	260 ± 60	850 ± 20	6.6 ± 0.4	16 ± 1

## Supplementary Materials

The following are available online at https://www.mdpi.com/2073-4360/12/1/183/s1, Figure S1. ^1^H NMR spectrum (400 MHz, D_2_O, 70 °C) of the purified sample PMAG-CTA after dialysis. Initial molar ratio [MAG]o:[CTA]o:[AIBN]o = 75:1:0.25, DMF, *T* = 70 °C, 16 h; Figure S2. Overlays of ^1^H-^13^C HSQC spectra of PMAG modified by AETL: 1—NaBH_4_ in DMF/water (50/50, *v*/*v*%), 2—NaBH_4_ in DMF, 3—Et_3_N + HexNH_2_, DMF; Figure S3. SEC traces of PMAG-CTA and PMAG modified by AETL (PMAG-NH_2_): (a) NaBH_4_ in DMF (sample #2), (b) Et_3_N+HexA; Figure S4. SEC traces of PMAG-CTA and PMAG-NH_2_ prepared by modification by AEMA; Figure S5. STEM images of PMAG-*b*-PGlu(OBzl) micelles, sample *b5*, (a, b) and PMAG-*b*-PIle polymersomes, sample *b8* (c). Scale bars: (a) 500 nm; (b) and (c) 1 µm; Table S1. Characteristics of amphiphilic PMAG*-b*-P(amino acid) determined by static and dynamic light scattering.
