# Supplementary material for: Bio-Inspired Amphiphilic Block-Copolymers Based on Synthetic Glycopolymer and Poly(Amino Acid) as Potential Drug Delivery Systems
Source: Polymers (Basel). 2020 Jan 10;12(1):183. doi: 10.3390/polym12010183 (PMC7023050; doi:10.3390/polym12010183)
Supplement: Supplementary file 1 [file polymers-12-00183-s001.pdf]

polymers

Supplementary Materials

Bio-Inspired Amphiphilic Block-Copolymers Based on Synthetic Glycopolymer and Poly(Amino Acid) as Potential Drug Delivery Systems

Mariia Levit[1], Natalia Zashikhina[1], Alena Vdovchenko[2], Anatoliy Dobrodumov[1], Natalya Zakharova[1], Anna Kashina[1], Eckart Rühl[3], Antonina Lavrentieva[4], Thomas Scheper[4], Tatiana Tennikova[2] and Evgenia Korzhikova-Vlakh[1,2]*

1 Institute of Macromolecular Compounds, Russian Academy of Sciences, St. Petersburg, Russia
2 Institute of Chemistry, Saint-Petersburg State University, St. Petersburg, Russia
3 Physical Chemistry, Institute of Chemistry and Biochemistry, Freie Universität Berlin, Berlin, Germany
4 Institute of Technical Chemistry, Gottfried-Wilhelm-Leibniz University of Hannover, Hannover, Germany

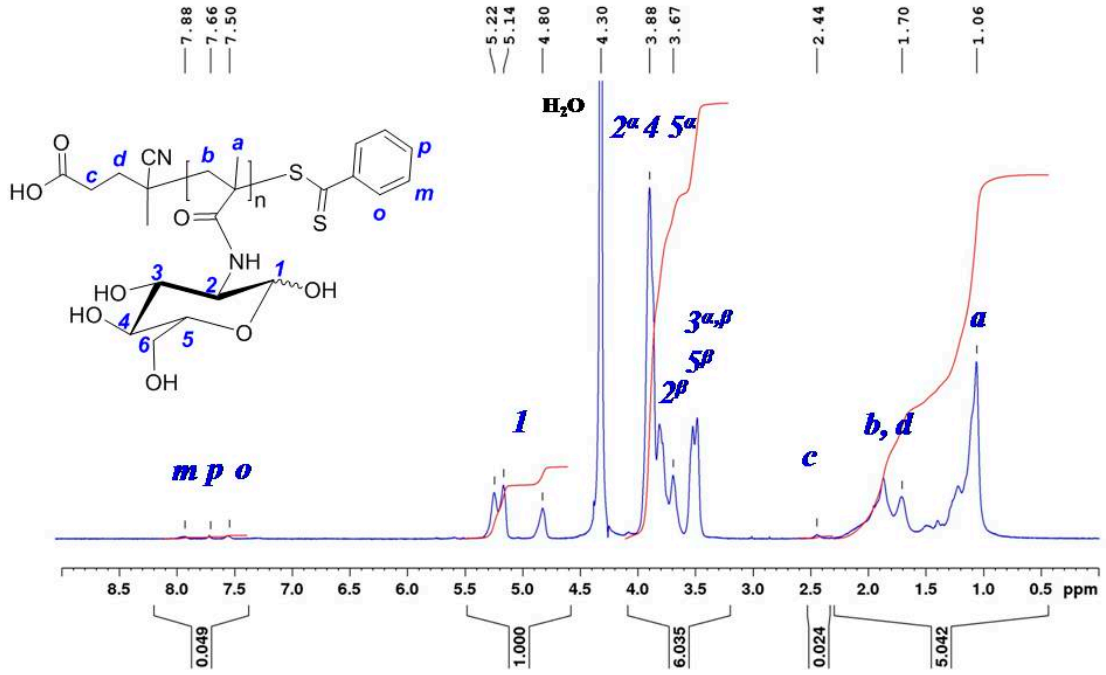

Figure. S1. ^{1}H NMR spectrum (400 MHz, D_2O, 70 °C) of the purified sample PMAG-CTA after dialysis. Initial molar ratio $[MAG]_0:[CTA]_0:[AIBN]_0$ = 75:1:0.25, DMF, *T* = 70 °C, 16h.

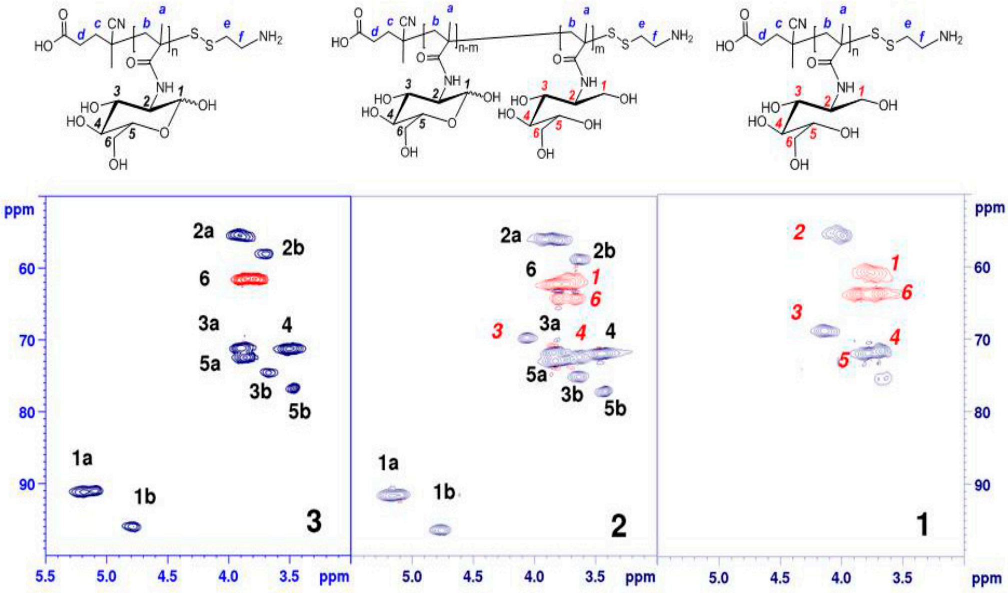

Figure. 2S. Overlays of ^{1}H-^{13}C HSQC spectra of PMAG modified by AETL:
1 – $NaBH_4$ in DMF/water (50/50, v/v%), **2** – $NaBH_4$ in DMF, **3** – Et_3N+$HexNH_2$, DMF.

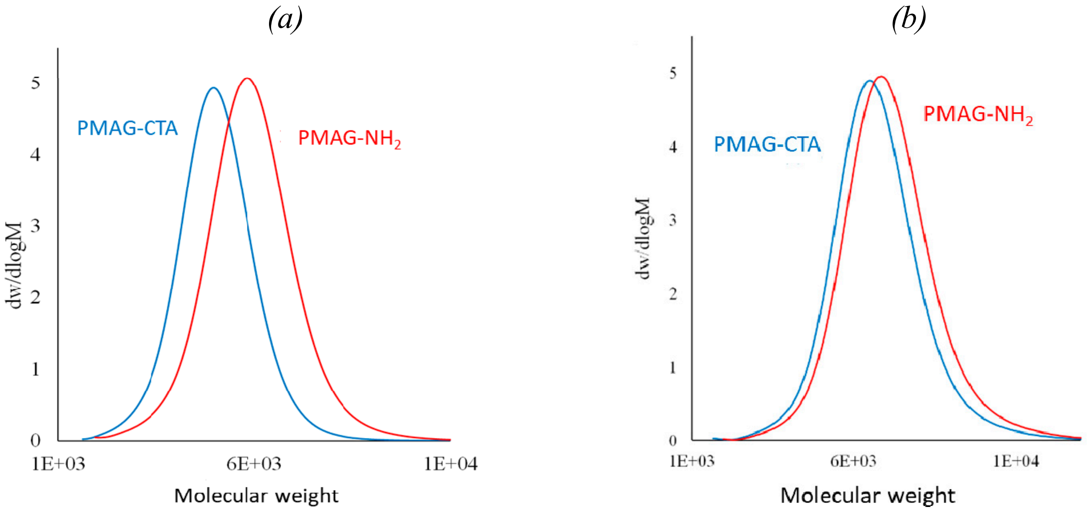

Figure S3. SEC traces of PMAG-CTA and PMAG modified by AETL (PMAG-NH_2):
(a) $NaBH_4$ in DMF (sample #2), *(b)* Et_3N+HexA.

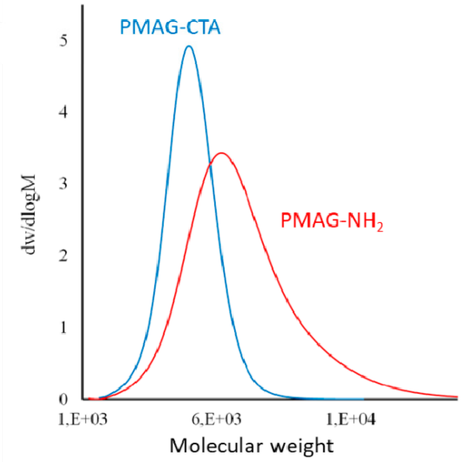

Figure S4. SEC traces of PMAG-CTA and PMAG-NH_2 prepared by modification by AEMA.

Table S1. Characteristics of amphiphilic PMAG-*b*-P(amino acid) determined by static and dynamic light scattering.

Sample #	dn/dc, cm^3/g	M_w	A_2, $cm^3 \cdot mol \cdot g^{-2}$	$R_{h\text{-}D}$, nm
b1	0.0740	5700	-1.56E-03	1.4
b2	0.0712	9000	-1.75E-03	1.7
b3	0.0706	10700	-2.16E-03	1.9
b5	0.0737	21400	-4.98E-05	3.2
b6	0.0787	26300	-6.38E-04	3.3
b7	0.0603	7700	-6.32E-04	≤ 1

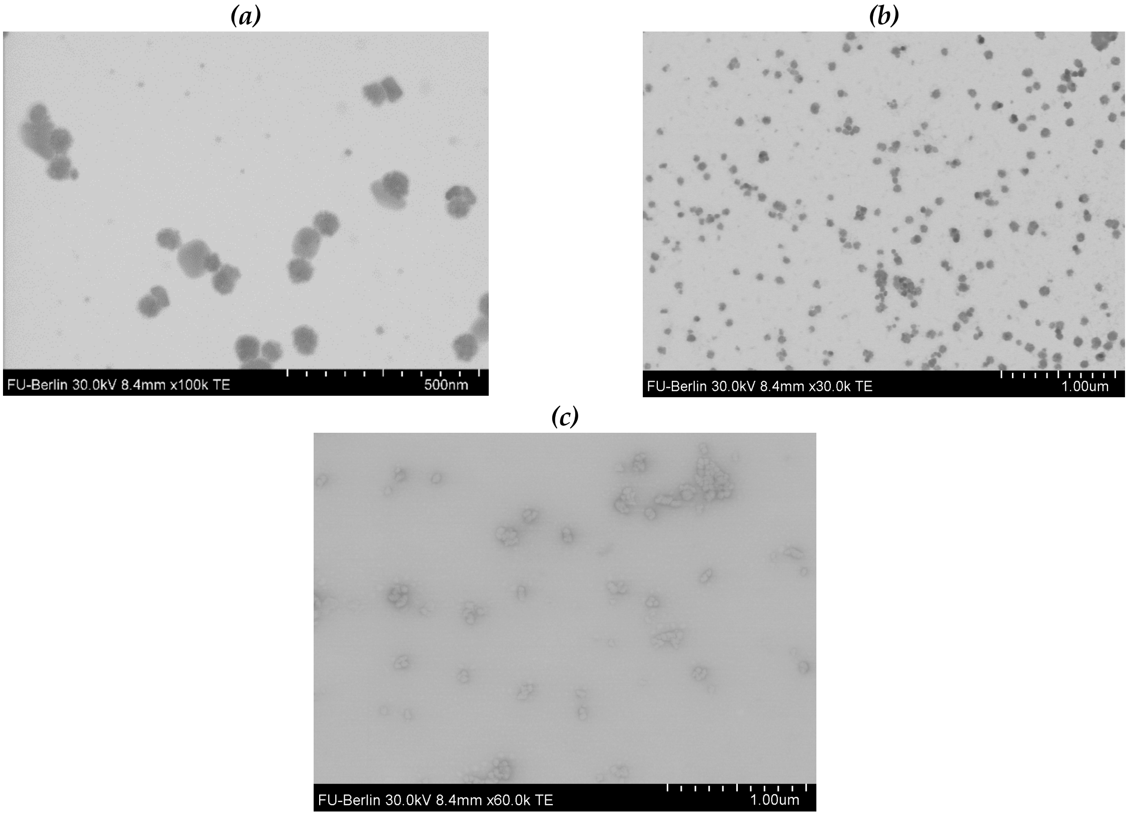

Figure S5. STEM images of PMAG-*b*-PGlu(OBzl) micelles, *sample b5,* ***(a, b)*** and PMAG-*b*-PIle polymersomes, *sample b8* ***(c)***. Scale bars: ***(a)*** 500 nm; ***(b)*** and ***(c)*** 1 μm.
